# Framing a Phenomenological Mixed Method: From Inspiration to Guidance

**DOI:** 10.3389/fpsyg.2021.602081

**Published:** 2021-03-03

**Authors:** Kristian Moltke Martiny, Juan Toro, Simon Høffding

**Affiliations:** ^1^Center for Subjectivity Research, Faculty of Humanities, University of Copenhagen, Copenhagen, Denmark; ^2^The Enactlab, Copenhagen, Denmark; ^3^Department of Sports Science and Biomechanics, University of Southern Denmark, Odense, Denmark; ^4^RITMO Centre for Interdisciplinary Studies in Rhythm, Time, and Motion, University of Oslo, Oslo, Norway

**Keywords:** phenomenology, mixed method, phenomenological interview, musical communication and absorption, research in cerebral palsy

## Abstract

Despite a long history of researchers who combine phenomenology with qualitative or quantitative methods, there are only few examples of working with a phenomenological mixed method—a method where phenomenology informs both qualitative and quantitative data generation, analysis, and interpretation. Researchers have argued that in working with a phenomenological mixed method, there should be mutual constraint and enlightenment between the qualitative (first-person, subjective) and quantitative (third-person, objective) methods for studying consciousness. In this article, we discuss what a framework for phenomenological mixed methods could look like and we aim to provide guidance of how to work within such framework. We are inspired by resources coming from research in mixed methods and existing examples of phenomenological mixed-method research. We also present three cases of phenomenological mixed methods where we study complex social phenomena and discuss the process of how we conducted the studies. From both the research inspiration and our own studies, we depict the landscape of possibilities available for those interested in mixing phenomenology with qualitative and quantitative methods, as well as the challenges and common pitfalls that researchers face. To navigate in this landscape, we develop a three-fold structure, focusing on (1) the phenomenological frame, (2) the phenomenologically informed generation of qualitative and quantitative data (tier one), and (3) the phenomenologically informed analysis and interpretation of data (tier two).

## Introduction

How do we investigate consciousness with its manifold nuances and complexities? Philosophers working within the philosophical tradition of phenomenology have, since its inception, tried to answer this methodological question, while breaking up disciplinary frontiers and working in interdisciplinary contexts. Despite this effort, there are fewer examples of working with a phenomenological mixed method. “Mixing” is here used as an umbrella term to refer to the multifaceted procedures of combining, integrating, linking, and employing multiple methods (Creswell, 2003; Creswell et al., 2003). Following Tashakkori et al. work, we define mixed-method investigations as “research in which the investigator collects and analyses data, integrates the findings and draws inferences using both qualitative and quantitative approaches” (Tashakkori and Creswell, 2007, 3). To work with a phenomenological mixed method is consequently to phenomenologically inform both the qualitative and quantitative data generation, analysis, and interpretation.

One of the few examples of a phenomenological mixed method is called “Neurophenomenology” as developed by Varela in relation to the proposal of naturalizing phenomenology (Varela, 1996; Varela and Shear, 1999). Varela argued that in order for cognitive science to work as a scientific method for studying consciousness, a mutual constraint [also called mutual enlightenment (Gallagher, 1997)] should exist between first-person (qualitative) and third-person (quantitative) methods in generating, analyzing, and validating both subjective and objective data. Philosophical phenomenology was used as the theoretical foundation for framing the mutual constraint and enlightening the two methods.

Guidelines of how to work with such neurophenomenological mixed methods have been developed under the heading of micro-phenomenology (Bitbol and Petitmengin, 2017). As we describe and discuss below, this way of working focuses on investigating the microdynamic processes and pre-reflective aspects of experience. Finding inspiration in micro- and neurophenomenology, we believe it is still necessary to show a variety of different ways to work with phenomenological mixed methods to be able to understand different kinds of experiences at different pre-reflective and reflective levels.

Our aim is to depict a landscape of possibilities available for those interested in this way of working. In this article, we will therefore discuss more broadly what a framework for phenomenological mixed methods could look like. In the attempt to develop a framework, we engaged ourselves in three studies to understand the process of working with phenomenological mixed methods.

The first study concerns the phenomenon of joint musical absorption. Having collaborated with the Danish String Quartet to establish definitions and criteria for various forms of joint musical absorption (Høffding 2019), we were interested to see if and how bio-rhythms were implicated herein. Would a strong sense of joint, “interkinesthetic absorption” (Høffding, 2019, 233-40) be matched by synchronized breathing and heart rate, or would physiology and phenomenology be divorced in this situation? This was the main research question driving our first mixed-method study. The methodological challenge we therefore had to solve was how we could investigate, both qualitatively and quantitatively, the experiences of musicians, while they were playing.

In addition to this study, we engaged in two mixed-method studies on physical disability. The first study focused on understanding joint actions involving people with cerebral palsy (CP), which is a disorder that occurs due to a non-progressive lesion in the developing central nervous system, damaging sensorimotor predictive models and processes and causing limitations to bodily functionality (i.e., bodily coordination and adjustments). In embodied and enactive accounts of cooperation which are phenomenologically informed, it is argued that there exists a strong and direct link between coordination in joint actions and the interactors' positive experiences of connectedness, harmony and flow (Fuchs and De Jaegher, 2009; Marsh et al., 2009; Fantasia et al., 2014). This means that people who have problems with bodily coordination, such as people with CP, would be expected to experience these positive feelings to a lesser degree, or not at all, making their experience of joint actions negative. Our research question was therefore: How do people with CP, who have problems with bodily coordination, experience positive and negative joint actions? To answer this question, we needed to design a mixed-method study where we could relate bodily coordination and positive or negative experiences for joint actions in the case of CP. However, this raised the methodological challenge of how to create positive and negative joint actions in a way where we could also investigate their functional and affective aspects?

The second study on physical disability focused on reducing prejudice toward people with physical disability. Most research on prejudice applies the Contact hypothesis (Binder et al., 2009). Its basic assumption is that contact—under appropriate conditions—will reduce prejudice and negative attitudes between in- and out-groups and between majority and minority group members—which in our cases means between people with and without physical disability. Inspired by the phenomenologically informed “interactive turn” in social cognitive science (e.g., de Jaegher et al., 2010; De Bruin et al., 2012; Schilbach et al., 2013; Satne and Roepstorff, 2015), our hypothesis was that embodied engagement as contact would reduce prejudice toward physical disability by changing the attitude to be more positive toward people with physical disabilities. To investigate this hypothesis, we needed a mixed-method design where we could test peoples' explicit (conscious) and implicit (unconscious) processes of attitude formation before and after an embodiment and engagement-based intervention. The methodological challenge we were dealing with was therefore: How do we change experiences of prejudice, while at the same time investigate the process of change?

In order to answer to these three challenges and provide a frame for phenomenological mixed methods, we need to review and develop a number of perspectives and analyses. In the next section Inspiration: Mixing With Phenomenology, we discuss what we mean by “phenomenological” in a mixed-method context, look at challenges found in the literature for working with phenomenological mixed methods, and discuss two inspirational examples, namely, microphenomenology (Bitbol and Petitmengin, 2017) and the EASE interview (Parnas et al., 2005) from phenomenological psychopathology. This inspiration was the point of departure from which we engaged in our three studies and which helped us in dealing with the methodological challenges, which we present in section Process: Three Cases of How to Use Phenomenological Mixed Methods.

In section Guidance: Steps, Decisions, and Standards, we combine the inspiration from the previous work with the lessons learned in conducting our studies. We develop and clarify a three-part structure that can serve as an overall guideline for the phenomenological mixed-method researcher. The structure consists of:

*The phenomenological frame:* The philosophical foundation, commitments, theories, concepts, and distinctions that frame two intricately linked tiers—starting from the theoretical point of departure and continuing through to the answers developed in response to the research question.
*Tier one:* The phenomenologically informed qualitative and quantitative data generation.
*Tier two:* The phenomenologically informed qualitative and quantitative data analysis and interpretation.

Although the three parts are presented as a chronological series of first, second, and third steps to take in order to conduct a phenomenological mixed method, the three parts are intricately linked and conducting phenomenological mixed-method research requires many steps back and forth between the three parts. We therefore do not believe that a procedure-like, step-by-step manual can be developed for researchers to follow within each of the three parts. As will be clear in the article, there is not one paradigmatic way to work with a phenomenological mixed method, but different ways in which qualitative and quantitative methods can be mixed within a phenomenological frame. Nevertheless, the aim of the three-part structure is to provide guidance and help those interested in phenomenological mixed methods take steps and make decisions that are performative and phenomenologically consistent.

## Inspiration: Mixing With Phenomenology

To understand what we mean by a phenomenological mixed method, it is useful to start by defining what is meant by “phenomenological” in a mixed method context.

### Phenomenological Foundations and Commitments

Fundamentally, the aim of phenomenology is to attain an understanding and description of the structures of human experience. It aims at being a rigorous science of consciousness, by pursuing “the things themselves” and taking experience seriously. This means letting the descriptions of conscious experience themselves come to the fore, withholding pre-established theories, explanations, and beliefs about the objects of conscious experience. It also means that phenomenology is opposed to the belief in the metaphysical realism that fuels various objectivist, scientistic, and naturalistic approaches (Gallagher and Zahavi, 2008). Metaphysical realism defends a mind- and experience-independent reality that can only truly and objectively be discovered by reducing and/or eliminating aspects of human experience and subjectivity. Such belief is, according to phenomenology, based on the objectivist illusion that our experience of worldly objects is irrelevant (or rather, an obstacle) when it comes to determining how objects really are, thus denying subjectivity any foundational ontological function, and further that it is possible to obtain a pure, absolute, and objective perspective on reality (Zahavi, 2017). In contrast, phenomenology argues that any understanding of the world comes from the first-person perspective of someone, even if this someone is a scientist who aims to take up an objective and third-person perspective in her research.

With such criticism of objectivism and naturalism, one might think that phenomenology would be better poised to include methods coming from qualitative research rather than quantitative research. One might further question whether it is at all possible to use phenomenology to mix both types of methods. In mixed-method research, this is called the problem of commensurability, namely, that mixing does not seem possible without contradiction, because the different methods reflect epistemologies and ontologies that are not compatible (Small, 2011).

In mixed-method research, scholars have not pointed to phenomenology as a solution to the problem of commensurability, but rather turned to pragmatism as their theoretical foundation (e.g., Rallis and Rossman, 2003; Johnson and Onwuegbuzie, 2004; Greene, 2007; Morgan, 2007; Denscombe, 2008). The basic principle in pragmatism for mixed methods is that the act of discovery should be prioritized over the theoretical justifications for knowledge. The researcher should apply whatever means and methods she finds useful for answering her research questions (Creswell and Plano Clark, 2011). However, in using phenomenology as a theoretical foundation for mixed methods, we neither need nor want to settle for a methodological strategy of “whatever works.” In fact, working with phenomenology is a way of prioritizing both the act of discovery and its theoretical justification.

Phenomenology argues that any understanding of the world comes from the first-person perspective of someone, but this does not mean that the aim is to develop a subjective account of experience. As with any scientific approach, phenomenology strives to avoid arbitrary or biased accounts of experience that focus solely and particularly on idiosyncratic experiences. Instead, phenomenology focuses on idiosyncratic experiences in order to understand and describe their invariant structures. Further, it emphasizes the interdependence of subjectivity and objectivity:

“the phenomenologists' focus on the first-person perspective is as much motivated by an attempt to understand the nature of objectivity, as by an interest in the subjectivity of consciousness. Indeed, rather than taking the objective world as the point of departure, phenomenology asks how something like objectivity is possible in the first place.” (Gallagher and Zahavi, 2008, 24)

Unlike more pragmatic, relativistic, and postmodern positions, phenomenology, at least on Gallagher and Zahavi's interpretation, does not deny the existence of objectivity, nor is it antiscientific, even if it surely questions the meaning of both objectivity and science. So this position does, indeed, poise phenomenology as a good starting point to mix methods that pertain to experiential or subjective aspects as found in qualitative methods and so-called objective aspects as found in the natural sciences and quantitative methods. This is also evidenced by the recent discussion on “naturalizing phenomenology” (Varela, 1996; Petitot et al., 1999; Varela and Shear, 1999; Zahavi, 2004).

In Høffding and Martiny (2016), we attempted to provide a framework for phenomenologically grounded work with interviews in qualitative research. Using this framework as an inspiration, the four principle commitments for working with a phenomenological mixed method are as follows:

1. To the thing itself: Using qualitative and quantitative methods to acquire detailed first-person and third-person understanding of an experience in question.
2. Invariant structures: Using qualitative and quantitative methods to grasp the invariant structures of the experience.
3. Subjectivity cannot be reduced to objectivity: In working with the qualitative and quantitative methods, the first-person perspective needs to be understood on its own terms, rather than reducing it to objective descriptions or deducing the qualitative from the quantitative.
4. Enaction, embodiment, and embeddedness: Phenomenology construes subjectivity and objectivity as embodied, enactive, and embedded. Qualitative and quantitative methods directly confront us with these aspects of experience.

Applying these commitments, we wish to maintain the complexity and irreducibility of conscious experience and the interdependence or co-constitution of subjectivity and objectivity. As emphasized in principle 4, this is done by understanding the research process in phenomenological mixed method as a social practice that rests on the enacted and embodied observations, experiences, and expertise of the individual researchers, but which is developed into shared knowledge of a research community through an intersubjective sense-making process (Depraz et al., 2003; Gallagher and Zahavi, 2008; Martiny, 2017). In other words, the research process includes a group of researchers with different perspectives based in either first-person, qualitative or third-person, quantitative science. Both of these perspectives are embedded and contextualized by the second-person, intersubjective perspective of the community. The aim of phenomenological mixed methods is to meaningfully integrate these three perspectives in order to make sense of the data and (hopefully) answer the research question.

### Front-Loading: Solving the Challenges of Ignorance and Hyperphilosophizing

Determining how to phenomenologically frame the research in the beginning of one's mixed-method research process is not without challenges. There is a spectrum between ignoring one's phenomenological point of departure and being overly and unproductively focused on it, in such a way that it leads to “hyperphilosophizing.”

In a recent and intense debate about how phenomenology should inform qualitative research debate, Zahavi has both criticized qualitative researchers for belittling and ignoring the contributions of phenomenologists like Husserl, Heidegger, and Merleau-Ponty (Zahavi, 2019b), and for hyperphilosophizing and misinterpreting their contributions such as the phenomenological method of epoché and the reduction (Zahavi, 2019a). Based on his critique, Zahavi suggests a productive and pragmatic way for qualitative researchers to work with a phenomenological point of departure. They should familiarize themselves with the phenomenological theory and its philosophical origin but refrain from focusing on its orthodoxy and directly adopting Husserl's, Heidegger's, or Merleau-Ponty's philosophical method by including methodological steps that are irrelevant for qualitative researchers such as epoché and the phenomenological reduction. They should therefore be informed by the comprehensive theoretical framework (ideas, concepts, and distinctions) that philosophical phenomenology has to offer, so that it makes sense in a qualitative research context and allows for better qualitative research results.

Zahavi and Martiny (2019) provide examples of how to pragmatically apply phenomenology in qualitative research. These examples—two of which we discuss below—can also be used as inspiration for phenomenological mixed methods. At the core of these examples, and one solution to the challenges of ignorance and hyperphilosophizing, is the idea of applying phenomenology by using phenomenological concepts, distinctions and theory, rather than its method.

One way to do this is to use the method of “front-loading,” which Gallagher (2003) originally proposed in order to work phenomenologically with experiments in cognitive science, and Køster and Fernandez (in press) recently proposed in order to phenomenologically ground qualitative research. More specifically, Gallagher writes about front-loading:

“Rather than starting with the empirical results (as one would do in various indirect approaches), or with the training of subjects (as one would do on the neurophenomenological approach discussed above) this third approach would start with the experimental design. The idea is to front load phenomenological insights into the design of experiments, that is, to allow the insights developed in phenomenological analyses (modeled on Husserlian description, or the more empirically oriented phenomenological analyses found, for example, in Merleau-Ponty, or in previously completed neurophenomenological experiments) to inform the way experiments are set up.” (2003, 91)

Taken in a mixed-method context, the idea is therefore to front-load concepts and distinctions from phenomenological analysis into the design of the qualitative and quantitative methods, and in this way theoretically frame the mixing of both methods. Throughout the article, we will give different examples of how to front-load phenomenology.

In Køster and Fernandez's (in press) own example, they front-load primarily Heideggerian, phenomenological concepts of “existentials” into their interview of people experiencing grief. These “existentials” refer to the essential structures of our being in the world, e.g., intentionality, selfhood, empathy, embodiment, temporality, spatiality, and affectivity. A quantitative example of front-loading is seen in cognitive science where the phenomenological distinction between “sense of agency” and “sense of ownership” is front-loaded into an experimental design using neuroimaging (Ruby and Decety, 2001; Chaminade and Decety, 2002; Farrer and Frith, 2002).

Being aware and taking responsibility for the phenomenological point of departure, i.e., commitments, concepts and distinctions, and front-loading it into the qualitative and quantitative methods, is therefore a necessary part of working with phenomenological mixed methods. Two of the most influential examples of how to work in such way comes from cognitive science and psychiatry.

### Phenomenological Mixed Method in Experimental Settings

As mentioned in the introduction, neurophenomenology is one of the few examples of phenomenological mixed methods. At the core of how neurophenomenology is conducted is the qualitative interview method called the “explicitation interview” (Vermersch, 1994)—nowadays renamed to the “micro-phenomenological interview.” What makes this interview method phenomenological is that Husserlian and other phenomenological ideas, distinctions (e.g., distinction between content and act of experience), and concepts (e.g., concepts of “pre-reflective experience” and “passive memory”) are front-loaded into the concrete interview techniques of generating and analyzing data of a micro-experience.

Practically, this means that the researcher uses open how-questions (how would you describe your experience?) in the interview to help the participants: (i) reenact a past experience, (ii) suspend their beliefs and theories about this experience, (iii) redirect their attention from the content of the experience to the appearance of this content, and (iv) come into contact with the pre-reflective dimension and microdynamic processes of the experience—which are usually unrecognized, unnoticed, or concealed (see Petitmengin, 2006 for a detailed clarification). In the analysis, it means that the Husserlian and phenomenological ideas, concepts, and distinctions are applied in analyzing the descriptions of the participants' particular and lived experience. From such analysis, it is then possible to discover generic structures of experience (see Petitmengin et al., 2019 for a detailed clarification).

In a phenomenological mixed-method context, the aim is to correlate the data and analysis from the micro-phenomenological interview with the data and analysis generated from quantitative methods. This can be done in different ways depending on the specific micro-experience under investigation, the research questions, and the aim of the correlation procedure. These aspects would influence when the qualitative and quantitative data are generated and what quantitative methods are used. Some examples of quantitative methods include working with brain imagery to investigate the experience of illusory depth perception (Lutz, 2002; Lutz et al., 2002) and the experience of seizures for people with epilepsy (Le Van Quyen and Petitmengin, 2002; Petitmengin et al., 2006, 2007), working with experimental protocols such as the Rubber Hand Illusion (Valenzuela-Moguillansky, 2013) and decision tasks (Petitmengin et al., 2013), and working with physiological and cardiac measures to investigate the experience of surprise (Depraz, 2018).

In summary of these examples, the mixed method of neurophenomenology can vary according to the following parameters (Bitbol and Petitmengin, 2017):

- Initiation: Starting with micro-phenomenological interview to identify experiential categories, or with quantitative measures to detect neuronal and physiological signatures.
- Mode of identification: Identifying the experiential variables before and “front-load” them into the experimental and quantitative design or using micro-phenomenological interview to gather phenomenological descriptions after the experiment.
- Level of temporal solution: Deciding at which time scale the correlation is looked for.
- Level of genericity: Is the correlation sought at the generic (type) level between experiential structures and neural-physiological signatures or at the token level between singular experiences and their specific neural–physiological correlates?
- Time analysis: If one wants to use new quantitative methods (e.g., intracranial Gamma-Band Mapping) to do real time analysis of the neuro-physiological signals and present the participants' and experimenters' with immediate (visual or auditory) feedback of the fine dynamics of this activity.

By reviewing two decades of literature on neurophenomenology, Berkovich-Ohana (2017) argues that neurophenomenology is appealing philosophically, but it is extremely difficult to implement experimentally, in both data generation and analysis. In some of the cases presented above, the method includes training the quantitative researchers and participants in Husserlian phenomenological methods such as epoché and phenomenological reduction. In neurophenomenology, there is therefore both the danger of “hyperphilosophizing” and that this mixed method primarily appeals to philosophers studying specific micro-experience. Micro-phenomenology has also been criticized for mis-representing phenomenology (Zahavi, 2011; Schmidt, 2018) and mis-construing a phenomenological understanding of the pre-reflective (Høffding and Martiny, 2016). That being said, it is nevertheless an increasingly popular and important method that begins to produce results and that we believe ought to influence and inspire phenomenological attempts at a mixed-method framework.

### Phenomenological Mixed Method in Clinical Settings

In clinical work on schizophrenia, two phenomenological interview protocols have been developed to supplement standardized diagnostic systems such as ICD-10 and DSM-5. These interviews are called the Examination of Anomalous Self-experience (“EASE”) (Parnas et al., 2005) and the Examination of Anomalous World Experience (“EAWE”) (Sass et al., 2017). These protocols front-load a primarily Husserlian phenomenology into a semistructured qualitative interview design and a semiquantitative psychometric checklist to generate data of patients' subjective experience.

As an example of how phenomenology is front-loaded into EASE and EAWE, both interviews proceed from the phenomenologically secured insight of the existence of a minimal, pre-reflective self-awareness and work with the proposal that schizophrenia could be centrally grasped as a disturbance of this minimal self-awareness. The phenomenological character of the EASE questionnaire can be directly inferred from many of its items, such as those in category 2, concerning “self-awareness and presence” (Parnas et al., 2005, 257). EASE also probes change in bodily experience (ibid), which can be seen as a continuation of the phenomenological insistence on the embodiment of subjectivity. EAWE further extends these insights, probing into changes in experience of the external world. This likewise flows out of a phenomenological orientation emphasizing the co-constitution of subjectivity and objectivity: certain changes in the experience of the world should reliably trace certain changes in subjectivity, found, in this case, in schizophrenia.

The motivation for working with such phenomenological mixed method is to obtain explanatory power in understanding, possibly diagnosing, and predicting schizophrenia, and thereby to expand the work on schizophrenia seen in the standardized diagnostic systems (Parnas and Henriksen, 2014), Henriksen et al. (under review). The interviews do so by prioritizing the patients' subjective experiences, which means that the focus is on the qualitative data of the patient's experience, which is generated while using the quantitative checklists and scoring sheets as a manual.

EASE and EAWE and their results have shown to be highly relevant both for diagnosis of schizophrenia and for the psychotherapeutic work involved in treating the condition. In relation to the latter, phenomenologically informed body-oriented psychotherapy (BPT) (Fuchs, 2005; Fuchs and De Jaegher, 2009; Fuchs and Schlimme, 2009; Koch and Fuchs, 2011; Fuchs and Koch, 2014; Fuchs et al., 2019) shows other ways to work with a phenomenological mixed method in schizophrenia research and clinical practice. In Galbusera et al. (2018), part of this work is conducted within an intervention framework where qualitative and quantitative methods are used to generate data sequentially before and after a BPT intervention. The methods for data generation include qualitative interviews, standardized symptom evaluation manuals, and Motion Energy Analysis software. They are used to understand and describe the therapeutic change processes for patients with schizophrenia using BPT, and the relation between the change processes and therapeutic results.

## Process: Three Cases of How to Use Phenomenological Mixed Methods

Based on the inspiration, challenges, and few cases of phenomenological mixed methods presented above, we engaged in three different studies to investigate complex social phenomena using phenomenological mixed methods.

### Understanding the Real-Life Experience of Joint Musical Absorption

Having established a phenomenological analysis of various forms of joint musical absorption (Høffding, 2019), we wanted to investigate the possible co-dependencies with heart-rate synchronization (HRS). Thus, concepts and distinctions from the phenomenological analysis were front-loaded into the design of the qualitative and quantitative methods.

The qualitative method used in the study was the approach of “phenomenological interview” where phenomenological analysis, commitments, ideas, and concepts are front-loaded into the interview design (Høffding and Martiny, 2016). The approach is a second-person, semistructured interview method with its own specific questioning and analysis techniques that use open “how” questions and specific strategies to co-generate detailed first-person descriptions of lived experiences. In this study, the interview focus was on how musicians experience their performance and how they experienced playing together.

Besides for HRS, there are many other biological and behavioral sources of synchronization we could have chosen to quantitatively investigate as co-determinants of joint musical absorption. Upham has conducted an activity analysis of music listeners' breathing synchronization and its coupling to music scores (Upham, 2018; Upham and Mcadams, 2018). Walton et al. have analyzed movement synchronization in music improvisation in a dynamical system framework (Walton et al., 2015, 2018); Swarbrick et al. have analyzed listeners' synchronized head bobbing (Swarbrick et al., 2019). Bishop et al. have analyzed both movement and eye-gaze synchronization in performers (Bishop et al., 2019), Bishop et al. (under review). In different settings, previous experiments have shown interesting correlations between personal relations and HRS in the context of shared experience in fire-walking ritual (Konvalinka et al., 2011) and choir-singing has also demonstrated strong couplings in heart rate (Müller and Lindenberger, 2011; Hemakom et al., 2016; Müller et al., 2018). We therefore chose to work with HRS.

The research strategy was consequently to cross-analyze the phenomenological interview data about the various experiences of joint musical absorption and the quantitative data measuring HRS (we used heart rate sensors produced by First Beat). To conduct the study, an interdisciplinary research team of biologists, psychologists, engineers, computer scientists, and phenomenologists collaborated with the musicians of “The Danish String Quartet.”

In the study, we wanted to include concert performances where musicians were playing, while we investigate the relation between their experiences and HRS. As you cannot interrupt a string quartet with questions about their sense of absorption, while they are performing, we needed to develop a way to generate the data. The following process was developed: The musicians were playing with heart rate sensors on their chest underneath their shirts and so the quantitative data was generated during the musical performance. The phenomenological interview data was generated after the concert performance. This particular process raised a methodological question about how to mix the two data sets in a coherent and rigorous manner. This methodological challenge derives from the limitations of the phenomenological interview: even though it can disclose some experiential richness of past specific moments reflected on, it is not designed to hold a 1-to-1 relation with quantitative measures down to the millisecond^1^.

To face this methodological challenge, we recorded sound (Zoom H5 or H6) and video (Garmin 360 virb) during the musical performance. The sound recording was played back to the musicians individually allowing them to evaluate their experience of absorption during the performance in a self-rating application: they listen to a recording of their recently played concert while rating with one finger on a tablet using a sliding scale going from “distracted” to “very absorbed.” This step ensures an automatic synchronization between the timing of the self-rating and the timing of the music. Moreover, since the recording of the heart rate is synchronized with the music in real time, we obtain a “bridge” to link the musicians' self-rated level of absorption and their heart rate.

After the musical performance, the musicians engaged in the self-rating sessions. The interviewing researcher could see the graphic representation of the results immediately after those sessions and used them to guide the qualitative interviews according to what was considered to be theoretically interesting. Already having in-depth knowledge of each of the DSQ musicians' phenomenology of absorption, relying on the ratings, Høffding could with a few questions ascertain how they experienced particular musical passages. In particular, he would enquire about each musicians' sense of the other ensemble members in selected moments, especially those of more intense forms of absorption. The data from the 360-degree video-recordings made it possible to contextualize the interpretation of the HRS, self-rating, and interviews. Among the relevant data provided by video-recordings were the musicians' behavioral and facial expressions.

**Figure 1:**
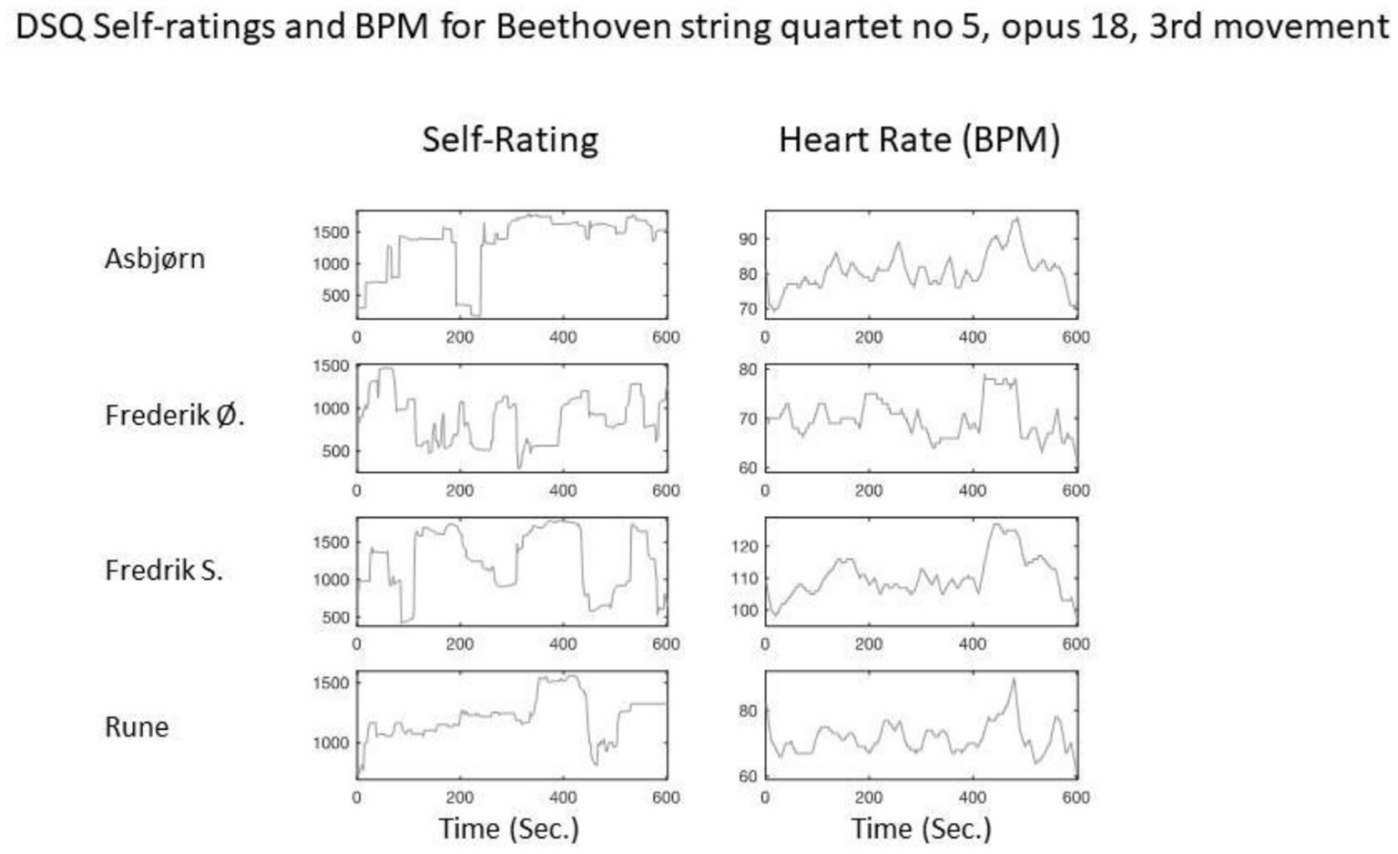
Graphs of post-concert self-ratings for absorption during a part of a particular concert in Denmark (left panel) and the associated HR profiles for each quartet member recorded during the actual concert. The y-axes represent (arbitrary) values on the rating scale, and heart beats per minute (BPM), respectively. The x-axes represent time in seconds. Note that the y-axes for each plot are scaled to the individual rating and HR curves for purposes of visual presentation.

Figure 1 shows an example of how the quantitative data was analyzed and visualized for one specific movement of a performance^2^. The similarities of beats per minute (BPM) across the musicians are clear in the graph, while the self-ratings of absorption are less homogeneous. Nevertheless, self-ratings converge around second 450, where a clear drop of absorption can be observed for two of the musicians (Rune and Frederik S.), and to a lesser degree for a third musician (Frederik Ø.). Just before this drop, there is also an absorption peak for all four musicians.

The qualitative data was analyzed in a descriptive manner. This meant that the recorded interviews were transcribed and from the transcription the data was coded and structured in order to identify experiential categories of joint musical absorption.

In the interpretation of the different analyses, the qualitative data analysis was contrasted with the quantitative data analysis for enriching the self-rating data. Here we see that two of the musicians describe that the drop in the absorption rating represents a transition from the fourth to the fifth variation in the third movement of Beethoven's 5th string quartet (opus 18), which is a compositional change from something intimate, slow, and *piano* to something much quicker, *forte*, merrier, and almost silly.

In quantitative terms, this development in the composition manifests itself in an increased amount of bodily motion, which can account for the BPM development in Figure 1. From the video recording, we see a break of around 0.5 s preceding the fifth variation, which enters in a sudden and energetic way after a clearly audible joint, deep, and fast in-breath. Two of the musicians reported the experience of “merryness” as associated with some level of ironic self-distance, which is absent in the intimate, *piano* variation preceding it. The violist, Asbjørn Nørgaard, agrees on the funny and almost silly character of the fifth variation. However, he does not link it to a drop in absorption since he considers himself equally absorbed in the funniness. These experiential reports help interpreting the different self-ratings for the three musicians. The fourth musician did not report a significant experience about the fifth variation.

The slightly contorted and even smiling faces of two of the musicians during those crucial seconds around the fifth variation, observable in the video recordings, contribute to contextualize the mixed analysis of the datasets. One might conclude that the change in the composition from the fourth to the fifth variation induces a drop in experienced absorption, which, perhaps not surprisingly, seems to generalize to the conclusion that musical genre and quality strongly impact the sense of joint absorption of the performers. Further, one can conclude that the self-ratings on their own can be deceptive and are best understood when constrained by interviews concerning those ratings.

Finally, as an exploratory pilot experiment, the current study also shows the complexity of integrating qualitative (interviews) and physiological (HRS) aspects of musical absorption. The fire-walking study (Konvalinka et al., 2011) that inspired the study on joint musical absorption showed a clear correlation between HRS and personal relations between actors and spectators in the ceremony. Part of its success was the relative simplicity of the task of crossing the burning coals coupled with the audience being stationary, all directly impacting the BPM and hence the HRS. When performing, all four musicians, however, are almost constantly moving at different paces: we need more research to identify ways to separate analyses of interview, self-rating, music score, quantity of motion, and HR before a meta-analysis and interpretation can more conclusively answer if heart rate synchronization plays a role in joint musical absorption.

### How to Create and Investigate Positive and Negative Experiences of Joint Actions in CP

In the study on joint actions involving people with CP, we wanted to answer the research question: how do people with CP, who have problems with bodily coordination, experience positive and negative joint actions? (Toro, 2020; Toro and Martiny, 2020), Martiny et al. (under review).

To answer this question, we needed to design a study where we could create positive and negative joint actions, investigate the data of bodily coordination and positive or negative experiences of joint interactions involving persons with CP, and then compare the data with similar data from interactions involving persons without CP (a control group). From previous phenomenologically informed qualitative work with CP (Martiny, 2015a,b), we knew that CP interactions are experientially different depending on who the people with CP are interacting with. So, we wanted to include CP interactions where one person with CP would perform joint actions with one person from three different groups of non-CP participants: (1) relatives, (2) therapists (stranger group #1), and (3) random strangers (stranger group #2). In the control setting, we included two persons without CP to perform the same joint actions.

We also knew from the previous work that everyday joint actions such as shaking hands, giving/receiving an object, or carrying an object together are very challenging for people with CP. So, depending on who the person with CP would interact with, the experience of such joint actions would be either positive or negative. In the experiment, we therefore developed six daily, hand-to-hand joint action exercises, which the two participants would perform sitting down at a table in front of each other.

In phenomenology, our mode of being-in-the-world is described as structured by our embodiment and primordially action-oriented. This means that corresponding with our worldly interests and our bodily skills and competences, we perceive objects as invitations for specific actions and we coordinate our bodily movement according to these perceptions (Merleau-Ponty, 2012). However, we also perceive other people as affording specific bodily responses and ways of engaging. This phenomenological analysis resonates well with Gibson's theory of affordances (Gibson, 1979).

Based on this analysis and theory, we therefore decided to track the participants' eye movements, as well as their bodily movements during the joint-action exercise. This quantitative data was generated using eye-tracking glasses (Tobii Pro Glasses 2) that the participants wore, so that both their eye focus and areas of interest were recorded. In addition, a Kinect (v1) camera was set up in the room to record 3D video data of the participants' bodily motion and movements.

The eye-tracking devices would also allow us to determine what regions of the environment seemed more attractive or relevant for the interactors during the interactions. We would then be able to analyze the predominant eye focus of the participants during the interaction—thus providing a signal of the person's attitude toward the interaction. For the idea of including the participants' attitudes in the analysis, we front-loaded Husserl's notions of personalistic and naturalistic attitudes (Husserl, 1989; Toro and Martiny, 2020).

Immediately after the joint action exercises were performed, we conducted a 15–20-min interview together with both participants to generate qualitative data of how the participants experienced the interactions. The interview was conducted using the approach of a “phenomenological interview,” as described above. In this study, the interview focus was on how participants with and without CP experienced the situation of acting together in the joint action exercises, how they experienced their own actions and the actions of the other participant, and how they experienced the interaction when functional challenges occurred.

To be able to conduct such a phenomenological mixed-method study, with both qualitative and quantitative data generation, we were an interdisciplinary team of philosophers, psychologists, and computer and cognitive scientists. For the analysis of the quantitative eye-tracking and bodily motion data, we imported the data and analyzed it using MATLAB. The quantitative analysis employed classical statistical models such as linear mixed-effects, full, and null models.

The quantitative analysis showed that during the interaction, the strangers looked significantly less at the face of the person with CP compared with relatives and therapists. The control group looked at each other's face much more than participants in the CP interactions and was also significantly faster in performing the exercises. Despite functional challenges, all CP interactions completed the exercises successfully and it took them approximately the same time. In the most demanding exercises, CP–stranger interactions were as quick or quicker than CP–relatives and CP–therapists. Also, in the CP–stranger and control groups the participants responded quicker to the facilitator's instructions and moved quicker toward the point of interaction, than CP–relatives and CP–therapists.

The qualitative data was analyzed in a descriptive manner. This meant that the recorded interviews were transcribed and from the transcription the data was coded and structured in order to identify experiential categories. The qualitative reports were categorized according to the participants' experience of the interaction in general (as positive or negative), their experience of the task, of the other person, of the situation, and of themselves. In the analysis, we found elements that allowed us to identify common categories of positive as well as negative experiences. Positive experiences were described as “natural,” “open,” “attuned,” “habitual,” and “calm.” Negative experiences were described with terms like “unnatural,” “alert,” “transgressive,” “functional,” “hesitant,” and “correct.” Overall, CP–stranger interactions were experienced negatively, while CP–relatives, CP–therapists, and controls had positive experiences of joint actions.

**Figure 2:**
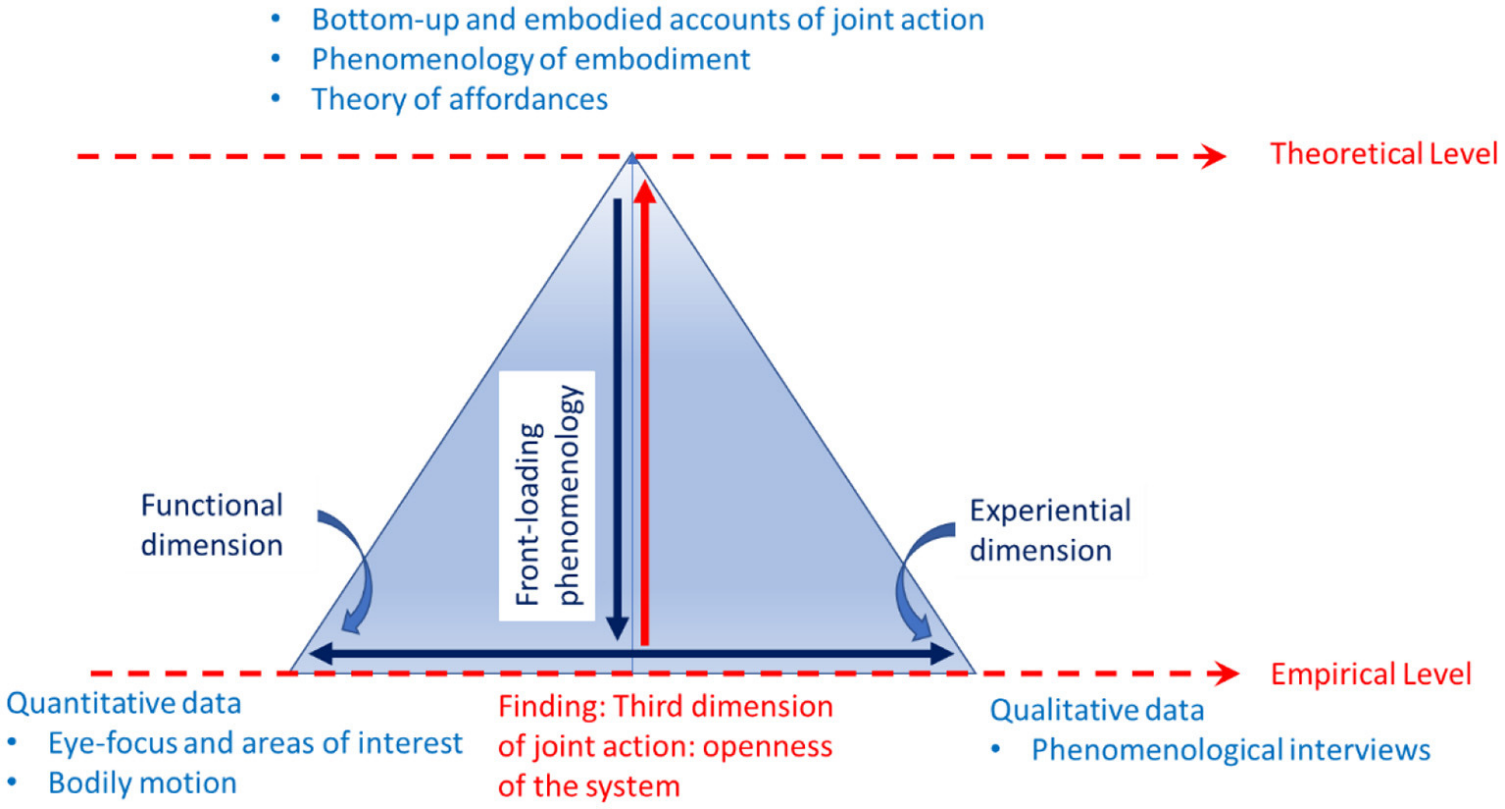
Diagram of the triangulation in the joint action experiment involving people with CP. The triangle depicts vertically the relations between the theoretical and empirical levels, and horizontally the relation between quantitative and qualitative data. At the theoretical level, the experiment was front-loaded by three main sources: (1) bottom-up and embodied accounts of joint action, (2) insights from the phenomenology of embodiment, and (3) the theory of affordances. At the empirical level, the relation between the functional and experiential dimensions is studied based on quantitative and qualitative data (horizontal blue arrow). From this analysis, an interpretation at the empirical level emerges, which suggests a different account of joint actions than the one offered by current theory (red vertical arrow).

In the two separate data analyses, we observed that most cases of CP–stranger interactions showed relatively high levels of coordination and goal accomplishment but were experienced as negative. We also observed that even though the CP–therapist and CP–relative interactions were functionally more challenging, they were experienced much more positively. To make sense of this complexity, we triangulated the two datasets with the front-loaded and a phenomenologically enriched theory of affordances to disclose the fundamental structures of the complexity of joint actions (see Figure 2).

The theoretical framework allowed us to mix and interpret the data into a unified account of the phenomenon of joint actions involving interactions with people with CP. Our findings contest current embodied and enactive accounts of joint actions, according to which there is a direct relation between the functional and experiential dimensions in joint actions. Our study showed the different ways that functionality and affectivity are interwoven in joint actions and how they are mediated by a third dimension of joint action. We propose that this third dimension is the openness of the system constituted by the participants in a joint action [see Martiny et al. (under review)].

Our proposal is phenomenologically inspired, as it reflects the complexity of social interactions—not only as relations between living—physical—bodies, objectively describable in terms of bodily coordination—but primordially, as a relation between lived bodies, embedded in socially and culturally rich contexts. Indeed, our experience of our own bodies, of the other person, of the situation, and of the interaction depends on more than just bodily coordination and performing specific tasks successfully.

### How to Change and Investigate Prejudicial Attitudes Toward Physical Disability

In the study of reducing prejudice toward people with physical disability, we wanted to test our hypothesis that embodied engagement as contact reduces prejudice toward physical disability by changing attitudes to be more positive toward people with physical disabilities. To test this hypothesis, we needed a design where we could investigate peoples' explicit (conscious) and implicit (unconscious) processes of attitude formation before and after an embodied engagement-based intervention.

As it turns out, contemporary ways of thinking about theater in theater and performance study is very much in line with phenomenologically informed social cognitive science. Theater is described by three notions, namely, (1) embodiment, (2) engagement, and (3) transformation. The notion of “embodiment” is used as a way of understanding the “affective” impacts that the performance has on the audience (Thompson, 2009; Nicholson, 2011), where the audience experiences “being kinaesthetically moved” (Fenemore, 2003). The notion of “engagement” is used to emphasize that in theater performance, performers and audience are in a shared, participatory, and immersed dialogue (Shepherd, 2006; Shaughnessy, 2012). The idea and notion of “transformation” describe how the engaged performance creates “effect” of social change through its embodied “affect” (Nicholson, 2005; Thompson, 2009). Thus, it seemed to us that theater would allow for embodied engagement and that it had the potential to create change.

A large team of both researchers (philosophers, psychologists, and researchers in performance studies and cognitive science) and practitioners (theater director, actors, dramaturgs, scenographer, and musician) collaborated to develop a specific embodied engagement intervention using a theater performance. The theater performance was developed as an autobiographical stage performance about a 28-year-old man, JN, who lives with quadriplegic cerebral palsy (CP) and has a speech impediment, thus displaying group salience both visibly and audibly at first encounter.

To measure the engagement-based intervention's effect on the audience, we wanted to investigate their attitudes toward physical disability before, during, and after the intervention. To do so, we designed a mixed-method research process consisting of both explicit (self-reporting) quantitative and qualitative methods, as well as implicit (behavioral) quantitative measures. The different methods were testing for both the successful achievement of embodied engagement and attitude change as part of the prejudice reduction.

The quantitative data was generated before and after using a 7-point Likert scale quantitative questionnaire and an Implicit Association Test (IAT), and it was generated at a restricted research test area close to the theater stage. The IAT test is a standardized test used to measure implicit attitudes, which means that we could not front-load phenomenological analyses, concepts, and distinctions into the test. However, the quantitative questionnaire was developed by choosing questions from previous quantitative questionnaires on physical disability and changing the way the questions was asked in order to correspond to the “how” questioning techniques coming from the phenomenological interview.

During the performance, an interactive questionnaire was also developed to generate quantitative data of the audience's behavior in forming their attitude toward physical disability. We wanted to see if their engagement toward JN on stage continued throughout the performance. So, we front-loaded the definition of engagement from Satne and Roepstorff (2015) into the design of an interactive questionnaire about JN, which was conducted within the actual performance. Satne and Ropestorff define engagement as an affective, emotional, and reciprocal we-experience in which one is committed to the other as a person. If the audience were personally committed to JN, we expected that they would then continue to answer the questions we ask them thought the performance. We therefore measured how long it took the audience to answer the questions of the interactive questionnaire, the number of audience members who answered, and whether they made answer revisions. The questions were designed based on concrete scenes that the audience had just experienced, and the answers were submitted on a 10-point Likert scale by using a mobile answering device that was placed in the participants' seats. The answers were shown in “real time,” anonymously on a projection wall on stage that everyone could follow.

Data from most of the audience (*n* = 2604) was generated using the interactive questionnaire, but only parts of the audience were recruited before (pre-group) and after (post-group) the performance and given the quantitative questionnaire and the IAT test. For comparison, a control group (*n* = 505) that did not see the performance was given the quantitative questionnaire. A focus group (*n* = 30) was also recruited and given the quantitative questionnaire and the IAT test after the performance. Fifteen participants of the focus group were chosen for follow-up qualitative interviews. The interview was conducted using the approach of “phenomenological interview,” as described above. In this study, the interview focused on how the audience experienced both JN on the stage and the theater performance in general and their experience of answering the interactive questionnaire.

The quantitative and qualitative data were analyzed separately. The quantitative analysis concerned statistical comparison of the data before, during, and after the performance. Here we employed classical statistical models such as a series Kruskal–Wallis H-test for the quantitative questionnaire, an independent-sample Mann–Whitney *U*-test conducted in SPSS for the IAT test, and the interactive questionnaire was analyzed descriptively.

The qualitative data was analyzed in a descriptive manner. This meant that the recorded interviews were transcribed and from the transcription the data was coded and structured in order to identify experiential categories. The qualitative reports were categorized according to three overall categories of experience: (1) the experience of the intervention, (2) experiences of a nuancing and reflective effect, and (3) experience of the attitude formation.

The qualitative data showed that the performance changed the audience's attitudes toward people with physical disability from being objectifying and prejudicial to being humanizing, personalistic, and inclusive. The interactive questionnaire showed that the audience was highly engaged during the performance, with a small amount of people in the audience that changed their answers. The IAT test showed no change in attitudes after the performance, whereas the quantitative questionnaire showed both significant positive changes in the attitudes of people in the audience as well as the effects of a decrease in positive attitudes.

The decrease in positive attitudes was surprising to us, since our hypothesis was that embodied engagement as contact would reduce prejudice toward physical disability by changing attitudes to be more positive toward people with physical disabilities. To understand this apparent conflict between our hypothesis and data, we used a triangulation strategy to develop a meta-interpretation of the different analyses.

The front-loaded and phenomenologically based, second-person theory of engagement was used to combine and mix the quantitative and qualitative datasets into one account of prejudice reduction. In this account, reduction is not seen as a matter of decreasing negative attitudes or increasing positive attitudes. In contrast to Contact theories, prejudice reduction in our account is understood as the nuancing of attitudes. In the performance, this nuance effect is initiated by a self-reflection process where people in the audience become aware of the act of forming their own attitudes. This attitude formation is highly influenced and intensified by the social setting of the theater.

Given this interpretation, we reformulated our initial hypothesis of focusing on positive attitude toward physical disability and presented this reformulation in terms of the Engage, Nuance, and Attitude formation (Enact) Hypothesis. The Enact hypothesis states that to change prejudicial attitudes, interventions should be designed so that persons involved become highly engaged with the attitude object (e.g., personalized outgroup member), engaging on both an embodied, affective, behavioral, and social level. Further, we suggest that the goal of prejudice reductions should not be thought of as changing either positive or negative attitudes, but as a nuancing of attitudes.

According to the Enact hypothesis, the reduction of prejudice occurs due to the increased embodied engagement with people with physical disability, which creates an explicit and conscious nuancing and self-reflective effect. This also explains the lack of significance in the IAT test, since such tests target “automatic” and “implicit” associations that operate at a lower (un)conscious level of attitude formation and change.

## Guidance: Steps, Decisions, and Standards

What does the inspiration from the previous work combined with the lessons learned in our studies mean for a researcher who wants to engage in phenomenological mixed methods? In this section, the aim is to collect the insights, provide guidance, and help those interested in phenomenological mixed methods to make consistent decisions through the different methodological steps.

As we have seen in all the different cases and examples, when working with phenomenological mixed methods, the first part is to clarify one's phenomenological frame and point of departure, i.e., commitments, theories, analyses, concepts, and distinctions. Here the steps to take and decisions to make regard how one will front-load phenomenology into one's research question and mixed-method design. The second part, as we have seen, concerns what this phenomenological frame and front-loading means for how we generate the qualitative and quantitative data (tier one). The third and last part concerns how the front-loaded phenomenology informs the analysis and interpretation of the qualitative and quantitative data (tier two).

In the following section, we will go through the three different parts, clarify the steps and decisions within each part, and end by discussing what defines consistency in the steps and decisions.

### The Framing: A Phenomenological Point of Departure

As argued above, phenomenology provides a good starting point to mix qualitative and quantitative methods. Here we can avoid the challenge of “hyperphilosophy” by front-loading phenomenology into the mixed-method design, instead of getting caught up in methodological orthodoxy.

The first step is therefore to use phenomenology to guide one's research question. This means that one should mix into the question both subjective (first-person) and objective (third-person) aspects of the experience one is trying to investigate. That could be questions including pre-reflective and microdynamic processes of experiences and their neural signatures, joint experiences (e.g., joint musical absorption and joint actions) and their corresponding bodily and physiological aspects (heart rate, eye gaze, and bodily motion), or experiences of prejudice and their implicit and behavioral components.

As we see in the different cases and examples, the specific motivations for why we want to investigate a specific experience differ. The next step is to clarify one's motivation, since the design of the phenomenological mixed method will vary according to the motivation and so will the interpretation of the data. Using Venkatesh et al. (2013) as inspiration, we can group the different motivations for working with phenomenological mixed methods into three categories:

1. *Strengthening:* An approach designed for using both qualitative and quantitative methods to strengthen the understanding of a specific experience. This is seen in the case of neurophenomenology, which uses qualitative and quantitative methods with two motivational reasons in mind:
  - Corroboration: To verify the findings from one type of data with data from the other type.
  - Compensation: To utilize one method and its data to compensate for the weaknesses of the other type of method.
2. *Improvement:* An approach designed for using both qualitative and quantitative methods to develop a richer understanding of an experience. This we can do either through:
  - Expansion: By utilizing the one type of study to expand the understanding of the findings of the other type. This is the motivation behind our study of musical absorption, where the aim is to expand already established qualitative analysis of joint musical absorption by measuring quantitative heart-rate data and cross-analyze both data sets.
  - Developmental: By using the one type of study to develop research questions, hypotheses, and understanding for the other type. This is seen in the case of EASE and EAWE where qualitative method and data (i.e., the patients' subjective experience) are prioritized in order to develop better understanding of schizophrenia within the quantitative, standardized diagnostic system.
3. *Holistic:* To use both qualitative and quantitative methods to develop a more holistic understanding of an experience, either through:
  - Complementarity/divergence: By using the mixed methods to gain complementary or divergent views on the same experience. This is the motivation behind our study of prejudice against physical disability, since we used both qualitative and quantitative methods to gain different views on prejudicial experience.
  - Completeness: To use the mixed methods to provide a complete picture of an experience. This is the motivation behind our study of joint actions involving people with CP, where we used both quantitative and qualitative methods to get a more complete picture of both the functional and affective aspects of joint actions.

After one has decided on either strengthening, improving, or providing a holistic understanding of a specific experience, the third and last step in framing one's research is to choose which type of phenomenological design to work with. In order to take this step, one must answer the following questions: What will be the significance of the strengthened, improved, or holistic understanding of the experience and why should one engage in phenomenological mixed methods for providing such understanding?

If the significance and scope relate primarily to the theoretical and experimental research field within which the research is done (e.g., research in joint actions), one can choose to work with a basic design. This can be a basic study and/or experiment, as we saw in our study of joint actions in CP, and which we saw in many of the mixed-method cases in neurophenomenology.

However, the design combination of phenomenology with qualitative and quantitative methods opens up possibilities for case studies and researching experiences in real-life contexts (e.g., musicians playing), for working with interventions (e.g., BPT intervention) and changing experiences (e.g., prejudicial attitudes) and for including the lived experience of patients to transform diagnosis and therapy (e.g., EASE and EAWE). Many of the productive examples of phenomenological mixed methods in health and clinical settings work with a transformative aspect, since they aim to improve or provide better therapy and healthcare (see Zahavi and Martiny, 2019; Toro and Martiny, 2020). If the significance and scope of one's research relates to case studies and interventions, including participants' lived experience (participatory research) or transformative matters, it makes it a type of advanced phenomenological design (Fetters et al., 2013).

After being clear on one's phenomenological point of departure, research question, motivation, and type of design, one has to figure out how to phenomenologically inform the data generation.

### Tier One: Phenomenologically Informed Data Generation

The first step in tier one is to clarify how phenomenology will be front-loaded into the qualitative and quantitative data generation. As we have seen, when it comes to the qualitative data generation this means working—in all the different cases and examples—with some version of the “phenomenological interview.” Depending on the experiences one is investigating, one can front-load different phenomenological analyses, concepts, and distinctions into the interview and generate the qualitative data in different ways. This means that there will be different possibilities for working with the interview that apply different framing, interview foci, questions, and techniques.

That being said, there is currently a methodological gap in how phenomenology is and can be front-loaded into other qualitative methods in a mixed-method context. What does it mean for phenomenology to be front-loaded into and inform for example: participant observations, video analysis, archival investigations, or discourse analysis? In Martiny et al. (2016), we discuss and give examples of how one can work phenomenologically with multimedia within qualitative methods and in our study of joint musical absorption we also used video analysis. However, to develop clear guidelines for how phenomenology could be front-loaded into a variety of qualitative methods would be a fruitful way to improve research within phenomenological mixed methods.

When it comes to the quantitative data generation, the decision is not about choosing one particular approach of how to front-load phenomenology into one quantitative method. Rather, as we have seen, there are many different examples of quantitative methods that phenomenology can be front-loaded into. For example, phenomenology can be front-loaded into the application of brain imagery, psychological tests and experimental protocols, quantitative questionnaires, standardized manuals, eye-trackers, bodily motion measures, motion energy measures, heart rate, and other physiological and cardiac measures. It is easier to make the decision about which quantitative methods to use by keeping the focus on the mixed aspect of the research. What is unique about phenomenological mixed methods is not which quantitative method one uses but how the qualitative and quantitative data generation processes are mixed and integrated into one's phenomenological research inquiry.

With inspiration from mixed-method research (Creswell et al., 2003), the next step to take in designing a mixed data generation process requires answering questions like: will the qualitative and quantitative data be generate more or less at the same time (concurrent) or in different phases, over a period of time (sequential)? Will the qualitative or quantitative data generation be prioritized as equally important, or will one be prioritized over the other, due to, e.g., practical constraints of data generation or the need to understand the one type of data before moving on to the other? How and when will the qualitative and quantitative data be integrated? By analyzing one data set before moving on to the next (connecting); by merging the two data sets in the interpretation; or by embedding one data generation process into the generation of the other datasets?

Depending on how one answers these questions, the data generation processes can be designed differently. As we see in neurophenomenology, one can start with the micro-phenomenological interview to help inform and/or interpret the quantitative data generation (e.g., brain imagery). One can also generate the quantitative data first and then conduct the micro-phenomenological interview afterward. In this way, the quantitative data will help inform and/or interpret the qualitative data. The first approach is an exploratory design, and the second is an explanatory design, but similar for both is having one research track where the data is generated sequentially and then connected to one another using the phenomenological frame.

In our studies of joint actions in CP and reduction of prejudice toward physical disability, we see that it is also possible to generate the qualitative and quantitative data more or less concurrently, i.e., during a similar timeframe. Here the phenomenological interview is used to generate the qualitative data and different methods (eye-tracking, bodily motion measures, IAT test, and questionnaires) are used to generate the quantitative data. Characteristic for such approach is that the two data generation processes are not dependent on one another. This means that one will have two tracks where the qualitative and quantitative data are generated in parallel. Using the phenomenological frame, the data can then be triangulated and merged in the interpretation of the data (tier two).

In EASE and EAWE, as well as in our study of joint musical absorption, we see that the qualitative and quantitative data generation is also done more or less concurrently. However, in the former, the qualitative data of the patients' experience are prioritized and the quantitative checklist and scoring sheets are applied as a manual for the qualitative interview. In the latter study, the phenomenological interview is embedded within a largely quantitative generation process. In this way of working with the mixed method, there will be one track of data generation where one of the qualitative and quantitative data processes crosses over and is embedded within the one. The phenomenological frame ensures that this crossover is theoretically coherent and therefore can occur.

**Figure 3:**
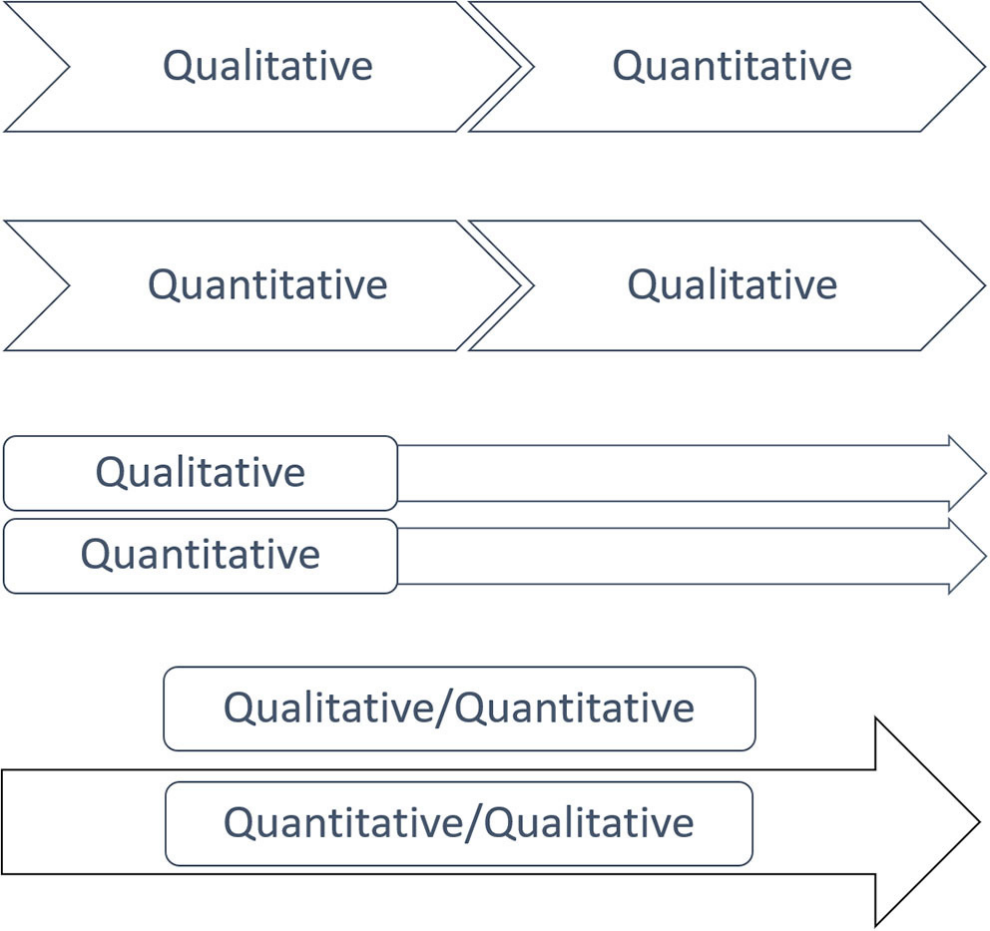
A description and illustration of the four types of data generation processes in phenomenological mixed method.

Using inspiration from mixed-method research (Creswell, 2003; Creswell and Plano Clark, 2007), we can categorize the following four major types of data generation processes, where the phenomenological frame integrates qualitative and quantitative data: Explanatory, exploratory, triangulation, and embedding (see Figure 3).

### Tier Two: Phenomenologically Informed Analysis and Interpretation

After having designed the data generation process, one must figure out how to analyze and interpret the data phenomenologically. Here, the first step follows from one's decisions in tier one. If one decided to generate the data sequentially (explanatory or exploratory) or in parallel (triangulation), the analysis of the qualitative and quantitative data will be done separately following either the sequential or parallel processes. This way of analyzing the data is seen in neurophenomenology and in our two studies on physical disability.

If one instead decides on embedding the data generation, the analysis will be one of conversion, where one form of data is converted into another form. This conversion can occur through quantification, as seen in EASE and EAWE, where the qualitative data (patients' experiences) are converted into quantitative data (numbers on a scoring sheet). It can also occur through qualification, as seen in our study on joint musical absorption, where quantitative data (self-rating) is converted into qualitative data (descriptions and narratives of the related experience). In our study of joint musical absorption, however, we also did separate qualitative and quantitative analyses, which means that this is a case of multimixed analysis, where one can use a combination of both separate and converted analyses in the process.

For the qualitative part of the data analysis—whether it is done separately or through conversion—phenomenology plays an important and explicit role. As seen in all the different cases and examples, the phenomenological analyses, concepts, and distinctions are applied in working with the recorded and transcribed interview data. The analysis process concerns coding and structuring the data in order to identify experiential categories and/or themes. For the quantitative part of the data analysis, we see different ways for phenomenology to inform the analysis in the cases and examples. The front-loaded phenomenological analyses, concepts, and distinctions help in deciding, for example, which quantitative data sample to focus on and which statistical models to use in analyzing this sample.

After having conducted the analysis, the next step is to interpret and make sense of the separated, converted, or multimixed analysis. one has for working with a phenomenological mixed method will influence this interpretation, since one will be looking for a strengthened, improved, or holistic understanding of a specific experience. This means that a fundamental part of the sense-making process in the interpretation includes keeping one's phenomenological frame, point of departure, and research questions in the foreground.

In addition to this suggestion, Teddlie and Tashakkori (2009, p. 289-293) provide some more general guidelines for mixed method sense-making. This includes separating one's research question into sub-questions, so that the relevant results for each sub-question can be summarized and examined. The questions can, for instance, be separated into the qualitative and quantitative research tracks (data and analysis). This exercise will provide some tentative interpretations and answers to the questions, which should then be mixed, i.e., compared, contrasted, combined, or the difference between them should be explained. The overall aim of this mixing is for the different interpretations and answers to the sub-questions to be integrated into one meta-interpretation.

For a phenomenological mixed method, the meta-interpretation should provide a generalized understanding of the structures of the experience in question (see commitments in section Phenomenological Foundations and Commitments). This means that although some partial interpretations and answers within the sense-making process might refer to particular experiences, the meta-interpretation should end up with generalized descriptions and understanding. As seen in the different cases and examples above, one is therefore able to both understand the experiences of particular patients, persons with disability and musicians, and what this means for understanding experiential structures in relation to, e.g., schizophrenia, joint actions, reduction of prejudice, and joint musical absorption. In a phenomenological mixed method, the interpretations and answers will therefore include a continuum of both particularity and generalization.

**Figure 4:**
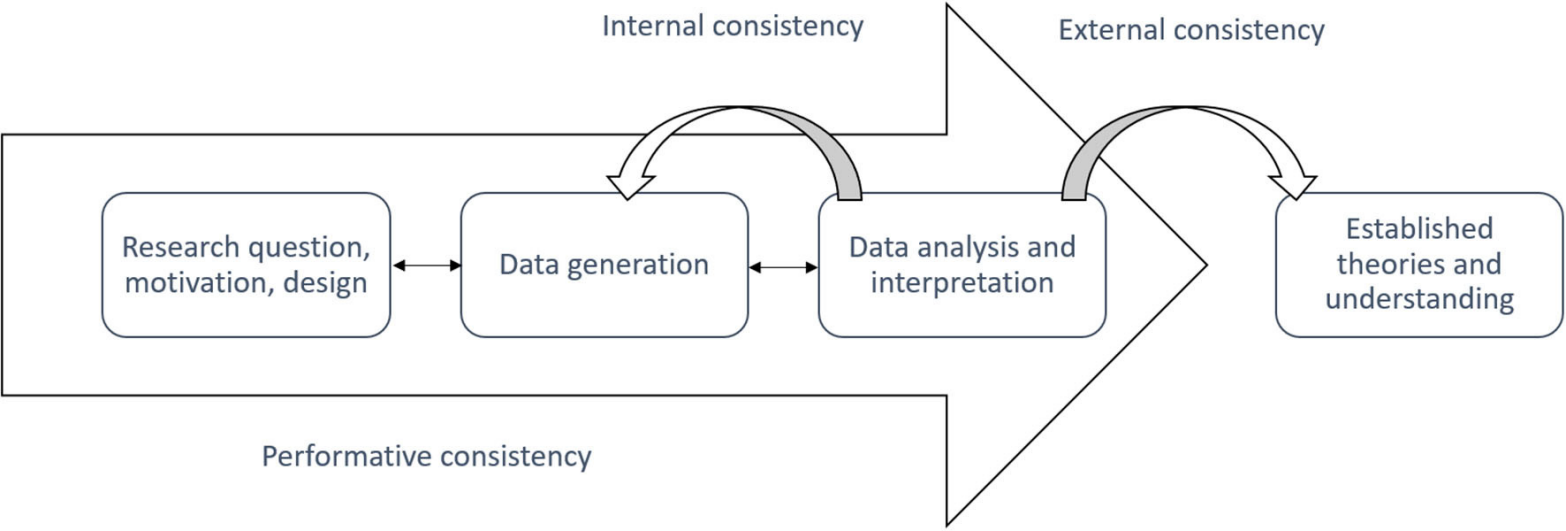
An illustration of the three forms of consistency in phenomenological mixed methods. Performative consistency refers to the consistency between the three parts of the phenomenological mixed method: Phenomenological frame, tier one, and tier two. Internal phenomenological consistency refers to how the interpretation relates to the data, and external phenomenological consistency refers to how the interpretation relates to already established theories and understanding of the investigated experience.

The meta-interpretation and the provided research answers of the phenomenological mixed method should be assessed in terms of the validity and quality of the sense-making processes. According to Tashakkori and Teddlie (2008) and Teddlie and Tashakkori (2009), this refers to the quality of the design and procedures, and the rigor and transferability (i.e., replication) of the interpretation and research results. In relation to a phenomenological mixed method, we propose that such validity and quality can be understood in terms of the “performative consistency” (Petitmengin and Bitbol, 2009) and the “phenomenological consistency” (Høffding and Martiny, 2016) of the sense-making process. This means that good phenomenological mixed-method research is conducted when there is a high degree of the following forms of consistency (see also Figure 4):

1. *Performative consistency:* The degree to which there is consistency between the three parts of the phenomenological mixed method, i.e., the phenomenological frame (commitments, theories, and concepts), tier one (the qualitative and quantitative data generation), and tier two (the mixed analysis and meta-interpretation).
2. *Internal phenomenological consistency*: The degree to which it is possible in the meta-interpretation to provide clear and coherent descriptions and explanations of the different qualitative and quantitative data and their relation.
3. *External phenomenological consistency*: The degree to which it is possible for the meta-interpretation to work with and against already established theories and understanding of the specific experiences in question. External phenomenological consistency is related to the methodological steps of “intersubjective validation” (Varela and Shear, 1999, 10) and “intersubjective corroboration” (Gallagher and Zahavi, 2008, 29–31).

In relation to neurophenomenology, Petitmengin and Bitbol describe “performative consistency” as a “validity in action,” which refers to the reproducibility of the method. They aim at prescribing a manual-like procedure for how to “correctly” conduct neurophenomenology. In Høffding and Martiny (2016), we acknowledge the overall idea of consistency between one's theories, methods, data, analyses, and interpretation but criticize the manual-like interpretation of performative consistency. In relation to phenomenological mixed methods, as we have seen in the different cases and examples, there is no one “correct” way to conduct the research, but many ways to mix methods phenomenologically. However, there should be a high degree of relational consistency between the three parts, i.e., one's phenomenological frame, how phenomenology is front-loaded and informs the methods used for data generation, and how the data is analyzed and interpreted based on the phenomenological frame and mixed methods.

This performative consistency will ensure that one's research can acquire a high degree of internal consistency. As we see in our three studies, for example, when complexity arises in comparing the qualitative and quantitative data or it seems that there are apparent conflicts between hypothesis and data, the phenomenological frame and theories can help in making clear and coherent sense of the data. In the two studies on physical disability, we also tried to create a high degree of external phenomenological consistency by not limiting the interpretations to already established theories of joint action and prejudice reduction. We also applied our joint action account in a rehabilitation research context to develop better-personalized healthcare for people with physical disabilities (Toro and Martiny, 2020) and develop a new theater performance for reducing prejudice for people with depression.

## Conclusion

In this article, we aimed at unearthing and making explicit important methodological considerations underlying a phenomenological mixed method, to guide researchers through the difficulties of studying experience using both qualitative and quantitative methods.

By framing the mixed-method research phenomenologically and beginning with a phenomenological point of departure, the research will proceed according to clearly established commitments that avoid the “whatever works” rationale and help the researcher to guarantee the consistency of their research.

As we propose, in applying the phenomenological frame one avoids “hyperphilosophizing” by front-loading phenomenology into the mixed method design, rather than getting caught up in methodological orthodoxy. This means that one should figure out what aspects of phenomenological analyses and theories are front-loaded, how they are front-loaded, and what this means for one's research question, motivation, type of design, data generation, analysis, and interpretation.

We have endeavored to show that there are different ways in which qualitative and quantitative methods can be mixed phenomenologically. We have developed the three-fold structure (the phenomenological frame, tier one, and tier two) for conducting phenomenological mixed-method research as a guideline through the landscape of possibilities available. The aim of the three-part structure is for those interested in phenomenological mixed methods to take steps and make decisions that are performatively and phenomenologically consistent.

## Data Availability Statement

The original contributions presented in the study are included in the article/supplementary material, further inquiries can be directed to the corresponding author/s.

## Ethics Statement

Høffding's study with the DSQ was reviewed and approved by the Norwegin Center for Research Data under number 613262. For the other studies, ethical review and approval was not required in accordance with the local legislation and institutional requirements. The patients/participants provided their written informed consent to participate in this study. Written informed consent was obtained from the individual(s) for the publication of any potentially identifiable images or data included in this article.

## Author Contributions

All authors listed have made a substantial, direct and intellectual contribution to the work, and approved it for publication.

## Conflict of Interest

The authors declare that the research was conducted in the absence of any commercial or financial relationships that could be construed as a potential conflict of interest.

## Footnotes

^1^In this regard, the micro-phenomenological method, with its capacity to describe short periods of experience with great nuance, might be better suited to link the reflected experience with the quantitative data.

^2^The raw data (audio recording, self-rating app scores, and the First Beat sensor data) is available at: https://www.uio.no/ritmo/english/news-and-events/events/musiclab/2021/dsq/.
